# Covalency in actinide(iv) hexachlorides in relation to the chlorine K-edge X-ray absorption structure†

**DOI:** 10.1039/d1sc06454a

**Published:** 2022-02-09

**Authors:** Dumitru-Claudiu Sergentu, Jochen Autschbach

**Affiliations:** Department of Chemistry, University at Buffalo State University of New York Buffalo NY 14260-3000 USA jochena@buffalo.edu

## Abstract

Chlorine K-edge X-ray absorption near edge structure (XANES) in actinide^IV^ hexachlorides, [AnCl_6_]^2−^ (An = Th–Pu), is calculated with relativistic multiconfiguration wavefunction theory (WFT). Of particular focus is a 3-peak feature emerging from U toward Pu, and its assignment in terms of donation bonding to the An 5f *vs.* 6d shells. With or without spin–orbit coupling, the calculated and previously measured XANES spectra are in excellent agreement with respect to relative peak positions, relative peak intensities, and peak assignments. Metal–ligand bonding analyses from WFT and Kohn–Sham theory (KST) predict comparable An 5f and 6d covalency from U to Np and Pu. Although some frontier molecular orbitals in the KST calculations display increasing An 5f–Cl 3p mixing from Th to Pu, because of energetic stabilization of 5f relative to the Cl 3p combinations of the matching symmetry, increasing hybridization is neither seen in the WFT natural orbitals, nor is it reflected in the calculated bond orders. The appearance of the pre-edge peaks from U to Pu and their relative intensities are rationalized simply by the energetic separation of transitions to 6d t_2g_*versus* transitions to weakly-bonded and strongly stabilized a_2u_, t_2u_ and t_1u_ orbitals with 5f character. The study highlights potential pitfalls when interpreting XANES spectra based on ground state Kohn–Sham molecular orbitals.

## Abstract 2

Chlorine K-edge XANES of An(iv) hexachlorides, calculated with multiconfiguration wavefunction theory, is interpreted in terms of similar metal–ligand covalency along the An = Th–Pu series.

## Graphical abstract

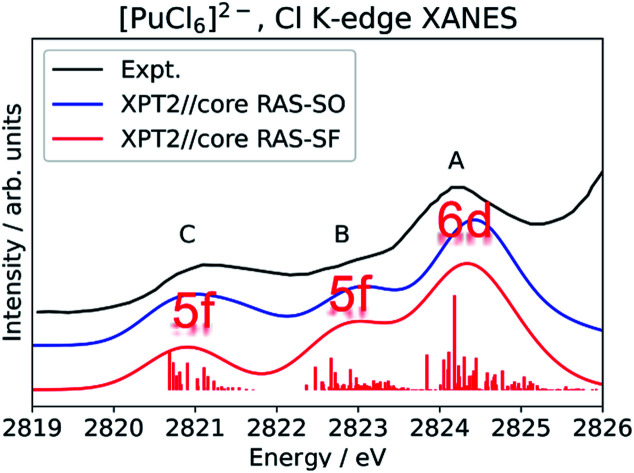

## 1 Introduction

Elucidation of the chemical bonding in lanthanide (Ln) and actinide (An) systems is presently the goal of many joint experimental and theoretical efforts. Such knowledge is of paramount importance for a wide range of applications, including the development of Ln and An-based single-molecule magnets,^1–6^ metal–organic frameworks,^7–11^ endohedral metallofullerenes,^12–17^ nanoparticles for industrial catalytic reactions and biomedicine,^18^ and the design of chelating ligands for f-element separation.^19–23^ Rational design of new f-element materials and separation ligands requires knowledge of the ionic *vs.* covalent behavior of the Ln 4f/5f and An 5f/6d shells. The development of computational tools for exploring the peculiar electronic structure of the heaviest elements is a key component of this research.^24^

With the rapid development of synchrotron X-ray techniques and predictive theoretical approaches, X-ray absorption near edge structure (XANES) spectroscopy, combined with theoretical calculations, has become a preferred tool to explore the local electronic structure of Ln and An ions in different chemical environments. Metal L_2,3_- and M_4,5_-edge and ligand K-edge XANES experiments and calculations have produced a wealth of knowledge about the oxidation state and metal–ligand bonding in a number of Ln and An systems that have long been of interest, *e.g.*, LnO_2_ (Ln = Ce, Pr, Tb),^25–34^ Ce(C_8_H_8_)_2_,^34–38^ An(C_8_H_8_)_2_ (An = Th, U),^39,40^ AnO_2_^2+^ (An = U–Pu),^41,42^ AnO_2_,^43–51^ Cs_2_UO_2_Cl_4_,^44,52,53^ LnCl_6_^*x*−^ (*x* = 2, 3),^54^ AnCl_6_^2−^ (An = Th–Pu),^55,56^ and others. A ligand K-edge measurement, for instance, probes bound core-excited states (ESs) arising from the ligand 1s core transition into unoccupied valence molecular orbitals (MOs) with varying degrees of metal atomic orbital (AO) contributions arising from hybridization, *i.e.*, metal–ligand covalent bonding. These transitions generate the pre-edge features seen in XANES spectroscopy, with intensity patterns and energetic band splittings that report the ligand-field splitting of the metal AOs and their contributions to the relevant valence MOs that are involved in the transitions.^57–61^

Through the years, efficient computational approaches have been developed for a fast and accurate prediction of XANES spectra in inorganic complexes of transition metals and f-elements. Finite difference methods and multiple scattering approaches^62–65^ are comparably fast in calculating such spectra, and reliable results were already reported for a variety of An complexes such as PuO_2_^2+^,^41^ PuO_2_,^48,50^ ThO_2_,^49^ and others.^66–69^ MO-based approaches for XANES calculations include the static-exchange approximation (STEX),^70,71^ time-dependent (TD) Kohn–Sham theory (KST, that is, KS density functional theory) with a restricted ‘excitation window’ (REW-TDKST),^71,72^ or simply ground state KST with the occupied and unoccupied MOs and their energies used in place of the many-electron states and their energies. These approaches were successfully employed in XANES calculations for the actinide systems UO_2_^2+^, OUN^+^ and UN_2_,^73^ AnCl_6_^2−^ (An = Th–Pu),^55,56^ AmCl_6_^3−^,^61^ U(C_7_H_7_)^−^_2_,^74^ and An(C_8_H_8_)_2_ (An = Th, U).^39^

Multiconfiguration (MC) self-consistent field approaches,^75–77^ often augmented with a treatment of the spin–orbit (SO) coupling *via* state-interaction, have become the preferred wave-function theory (WFT) tools for calculating XANES spectra of f-element complexes. An obvious reason is that MC-WFT with appropriately chosen active orbital spaces is capable of dealing with the (static) electron correlation generated by unpaired electrons distributed among near-degenerate orbitals. Another advantage over KST methods is that WFT calculations can be improved systematically, at least in principle. Unless it is necessary to distinguish, for example, between restricted or complete active space (RAS and CAS) types of calculations, we refer to these methods collectively as MC-WFT. Such calculations have been used during the past decade to treat core excitations in transition metal complexes.^78–93^ Inspired by these studies and ref. 94, in 2018 we started to explore the potential of MC-WFT with orbital optimization for the core-ESs for predicting accurate XANES spectra in f-element systems. So far, we have successfully calculated and analyzed the An M_4,5_ edges of AnO_2_^2+^,^42^ the Cl K-edge of AmCl_6_^3−^,^42^ the An N_4,5_ edges and C K-edge of An(C_8_H_8_)_2_ (An = Th, U),^40^ the C K-edge of U(C_7_H_7_)^−^_2_,^74^ and the Ce L_2,3_ edges of CeO_2_ and Ce(C_8_H_8_)_2_.^34^ In ref. 34, we were recently able to rationalize the notorious two-peak feature of the Ce L_3_ edge that puzzled the community for decades, in terms of metal oxidation states and multiconfigurational nature of the ground state (GS) *vs.* the core ESs as determined by *ab initio* calculations.

The measured spectra being what they are, their assignment and interpretation in terms of chemical bonding are always derived with the help of theoretical models based on calculations performed at varying levels of theory. The interpretation of the spectra may then depend implicitly or explicitly on the approximations made in these calculations. The observed intensities probe the GS and the core ESs simultaneously, by means of the transition moments. In MC-WFT, the ES wavefunctions are calculated explicitly, which offers the opportunity of being able to analyze chemical bonding in the GS and the ESs individually. A drawback is that the dynamic correlation in MC-WFT is typically treated as a correction to the state energies but not reflected in the wavefunctions themselves. Nonetheless, we found previously that even with minimal active spaces, calculations based on RAS wavefunctions with or without state energies corrected for the dynamic correlation can render the interpretation of XANES spectra very intuitive in chemical terms, because they inherit part of the differential correlation between the ESs and the GS in terms of orbital relaxation.^34,42^ In other words, these calculations are able to describe the transition not only in terms of changes in the MO occupations, but also in terms of changes in the MOs in cases where the chemical bonding in the GS and the ESs differs significantly. The interpretation of some XANES spectra may depend on the latter aspect,^34^ whereas for other spectra orbital relaxation may not be important.

In an effort to develop MC-WFT further for the prediction and analysis of An XANES spectra and the analysis of chemical bonding in the various electronic states, we decided to study the Cl K-edge XANES of AnCl_6_^2−^ with An = Th, U, Np, and Pu. Experimental spectra were reported by Su *et al.*,^56^ along with KST and TDKST calculations for analysis. For An = U–Pu, REW-TDKST calculations by Govind and de Jong^55^ reproduced/predicted the experimental spectra well. One of our aims is to show that MC-WFT likewise predicts the Cl K edges accurately. Another aim is to revise previous conclusions that were drawn regarding the extent of An(5f)–Cl(3p) covalency in relation to the XANES peak patterns and trends. Specifically, there is no need to draw a distinction between ‘covalency’ caused only by near-degeneracy of the participating fragment orbitals, *versus* both AO overlap and energetic match causing covalent bonding as it is traditionally explained in MO theory. We show that a previously assigned increased 5f covalency from Th and U to Pu is not required to rationalize the K-edge XANES spectrum peak intensities. It is demonstrated that these spectra reflect similar metal–ligand covalent bonding in the series. The actinide 5f shell stabilization with increasing actinide atomic number leads to the appearance of additional pre-edge features. The present study provides means of interpreting the chemistry of actinide 5f and 6d shells in the context of XANES spectroscopy, from the perspective of MO theory concepts that are familiar to chemists.

## 2 Computational details

The electronic structures and Cl K-edge XANES of [AnCl_6_]^2−^ (An = Th–Pu) were calculated with MC-WFT using OpenMolcas.^95–97^ As in the previous KST/TDKST study,^56^ octahedral geometries were employed, with average An–Cl bond lengths determined from the experimental structures of Z_2_[AnCl_6_] salts (Z = PPh4^+^ or NMe4^+^).^56,98–100^ Note that these geometries led to similar TDKST-calculated XANES spectra as did the slightly distorted experimental geometries that do not exhibit the inversion symmetry, or the geometries fully optimized in periodic molecular dynamics calculations.^55^ In the K-edge spectra, the intensity of dipole–forbidden transitions would of course be extremely sensitive to even slight distortions by removing the inversion symmetry center. However, for dipole-allowed transitions, minor modulations of intensity upon slight distortions are expected to be within the noise of the calculations and the experimental resolution. Single-point calculations were carried out in the *D*_2h_ abelian subgroup of *O*_h_. Sets of spin-free (SF) CAS and RAS self-consistent field (CAS/RASSCF)^75–77^ wavefunctions were calculated for the different spin multiplicities generated by the 5f configurations of the metals. Subsequently, SO coupling was introduced *via* the state interaction^101^ among the SF states (RASSI), using atomic mean-field integrals^102^ to construct the SO Hamiltonian. Chlorine K-edge XANES spectra were calculated with the core-RAS approach following similar strategies as devised in our previous work.^34,40,42^ Details regarding the selection of the active spaces and numbers of calculated valence and core ESs are provided in Section S1 in the ESI.† The calculated spectra were generated with Gaussian broadening for the individual transitions of *σ* = 0.45 eV (SF) and 0.40 eV (SO). The calculated energies were shifted by comparatively modest amounts, as listed in Table S1,† and scaled to align the prominent peaks with the experimental spectra for better comparison. Scalar-relativistic effects were treated by the second-order Douglas–Kroll–Hess Hamiltonian.^103–106^ All-electron atomic natural orbital relativistically contracted Gaussian-type basis sets^107^ of valence triple-*ζ* quality (ANO-RCC-VTZP, without *h* functions for An) were used for all atoms. When attempting to treat the dynamic correlation by RAS second-order perturbation theory (PT2),^108,109^ we encountered problems with symmetry breaking. Dynamic correlation effects on the SF state energies were treated instead with series of individual single-state extended multistate (XMS) PT2 ^110^ (XPT2) calculations for individual or groups of degenerate states. The XPT2 calculations used an ionization-potential-electron-affinity (IPEA) shift^111^ of zero and an imaginary shift^112^ of 2 au in order to eliminate intruder states and to produce similar reference weights in the valence and core ESs. The shells below 5d (An) and 2p (Cl) were kept frozen in the XPT2 calculations.

Accompanying scalar relativistic KST calculations were conducted with the Amsterdam Density Functional program (ADF, v2019),^113^ in the spin-restricted (RKS) and spin-unrestricted (UKS) fashion, using fractional occupations for degenerate orbitals. Unlike UKS, which produces separate sets of α- and β-spin orbitals, RKS generates a single set of orbitals that turned out to be similar to the set of orbitals generated from the MC-WFT calculations, thus enabling a straightforward comparison. The global hybrid PBE0 ^114,115^ functional was used in conjunction with all-electron doubly polarized triple-*ζ* (TZ2P) Slater-type orbital (STO) basis sets,^116^ the scalar relativistic zeroth-order regular approximation (ZORA) Hamiltonian,^117^ and an ‘excellent’ setting for the numerical integrations with Becke grids.^118^ To maintain consistency with ref. 56, where KST calculations with ADF were reported as well, the most diffuse Cl 3s and the most diffuse An 6d basis functions were removed to avoid negative Mulliken AO populations.

The GS and various SF core ES wavefunctions representative of the Cl K-edge XANES were subjected to quantum theory of atoms in molecules (QTAIM)^119,120^ analyses to uncover trends in chemical bonding and differences between the GS and the core ESs. QTAIM analyses of RAS wavefunctions were carried out with Multiwfn,^121^ while QTAIM analyses of KST/PBE0 electronic structures were carried out with the ‘Bader’ module of ADF. Natural Bond Orbital (NBO) calculations and natural population analyses (NPA) were carried out with the NBO6 program.^122,123^ The Molden2AIM^124^ package was used to generate NBO inputs and extract Mayer bond orders (MBOs) from the CAS/RAS wavefunctions. Wiberg bond orders (WBOs) were obtained from the NBO calculations.

## 3 Results and discussion

The average An–Cl equilibrium distance^56,98,99^ in [AnCl_6_]^2−^ is 2.681 Å (Th), 2.621 Å (U), 2.596 Å (Np) and 2.566 Å (Pu). The overall bond-length contraction from Th to Pu (0.115 Å) is somewhat smaller than the contraction in the radii of six-coordinate An^4+^ ions (0.19 Å) indicating that the metal–ligand interactions are not purely ionic. The octahedral ligand field splits the An 6d orbitals energetically and mixes them with ligand combination of matching symmetry to produce e_g_ (An–Cl σ and σ*) and t_2g_ (π and π*) MOs. The An 5f orbitals mix with ligand combinations to form a_2u_ (δ nonbonding), t_2u_ (π and π*) and t_1u_ (mixed σ and π bonding and antibonding) MOs. The antibonding MOs are mostly metal centered, and together with the nonbonding a_2u_ they are the MO theory counterparts of the actinide 6d and 5f orbitals from crystal field (CF) theory.

### 3.1 Ground-state electronic structure

**Fig. 1:**
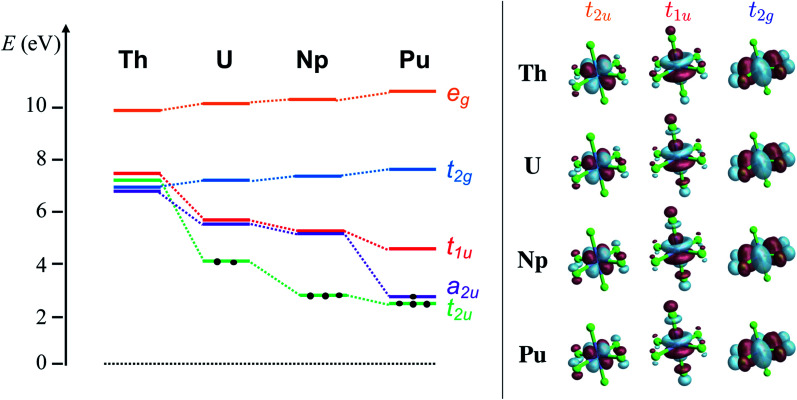
RKS/PBE0 calculations. Left panel: MO diagram depicting the relative energies of the An-centered 6d e_g_ and t_2g_ and 5f a_2u_, t_2u_ and t_1u_ orbitals of [AnCl_6_]^2−^ (An = Th–Pu) systems. Right panel: Isosurface plots (±0.03 au) of the 5f t_2u_, t_1u_ and 6d t_2g_ MOs with An–Cl antibonding character.

The KS MO diagram and isosurface plots of relevant KS MOs are shown in Fig. 1. GS electronic structure details such as configuration, metal valence f and d populations, *n*_f_ and *n*_d_, respectively, from NPA and Mulliken analyses, and Mayer/Wiberg bonds orders (MBO/WBO), obtained with RKS/PBE0 and CAS/RAS approaches, are collected in Table 1. QTAIM metrics are provided in Table 2.

**Table 1:** Calculated SF GS configurations (cfg.) with various approaches, f-shell (*n*_f_) and d-shell (*n*_d_) populations from NPA/Mulliken analyses, and Mayer/Wiberg bond orders (MBO/WBO) for [AnCl_6_]^2−^ (An = Th–Pu)

An	Aproach	GS cfg.	*n*_f_ a	*n*_d_	MBO/WBO
Th	CAS(12, 13)	100 a^0^_2u_t^0^_2u_t^0^_1u_	0.80/0.69	1.35/1.12	0.66/0.73
	HF	a^0^_2u_t^0^_2u_t^0^_1u_	0.78/0.66	1.35/1.13	0.66/0.73
	core-RAS^b^	a^0^_2u_t^0^_2u_t^0^_1u_	0.78/0.66	1.35/1.13	0.66/0.73
	RKS/PBE0	a^0^_2u_t^0^_2u_t^0^_1u_	1.03/0.91	1.70/1.55	0.99/0.89
U	CAS(14, 13)	60 t^2^_2u_ + 34 a^1^_2u_t^1^_2u_	2.89/2.59	1.43/1.18	0.65/0.77
	CAS(2, 7)	60 t^2^_2u_ + 35 a^1^_2u_t^1^_2u_	2.85/2.55	1.43/1.19	0.65/0.77
	core-RAS^b^	60 t^2^_2u_ + 35 a^1^_2u_t^1^_2u_	2.85/2.55	1.43/1.19	0.65/0.77
	RKS/PBE0^c^	t^2^_2u_	3.35/3.05	1.78/1.60	1.04/0.98
Np	CAS(15, 13)	57 t^2^_2u_t^1^_1u_ + 36 a^1^_2u_t^2^_2u_	3.79/3.52	1.47/1.19	0.63/0.76
	CAS(3, 7)	59 t^2^_2u_t^1^_1u_ + 34 a^1^_2u_t^2^_2u_	3.76/3.49	1.48/1.19	0.63/0.76
	core-RAS^b^	59 t^2^_2u_t^1^_1u_ + 34 a^1^_2u_t^2^_2u_	3.76/3.49	1.48/1.19	0.63/0.76
	RKS/PBE0^c^	t^3^_2u_	4.36/4.09	1.79/1.58	1.04/0.95
Pu	CAS(16, 13)	44 a^1^_2u_t^1^_2u_t^2^_1u_ + 38 a^1^_2u_t^3^_2u_ + 16 t^2^_2u_t^2^_1u_	4.68/4.49	1.56/1.23	0.64/0.72
	CAS(4, 7)	44 a^1^_2u_t^1^_2u_t^2^_1u_ + 39 a^1^_2u_t^3^_2u_ + 17 t^2^_2u_t^2^_1u_	4.65/4.46	1.57/1.24	0.64/0.72
	core-RAS^b^	44 a^1^_2u_t^1^_2u_t^2^_1u_ + 39 a^1^_2u_t^3^_2u_ + 17 t^2^_2u_t^2^_1u_	4.65/4.46	1.57/1.24	0.64/0.72
	RKS/PBE0^c^	a^1^_2u_t^3^_2u_	5.37/5.14	1.77/1.54	1.03/0.94

a Following ref. 125 and 126, *n*_f_ counts 5f and 6f AO populations (see Table S2).

b In addition to CAS(*n*, 7), core RAS includes the Cl 1s orbitals with at most one hole allowed, in RAS1, and the 6d t_2g_ orbitals with at most one electron occupation allowed, in RAS3. CAS(*n*, 7) and core RAS are of identical quality w.r.t. the GS.

c UKS/PBE0 data are provided in Table S3.

**Table 2:** QTAIM metrics calculated at the An–Cl bond critical points (BCPs) of the [AnCl_6_]^2−^ (An = Th–Pu) complexes. SF calculations

Approach	An	*ρ* ^bcp^	∇^2^*ρ*^bcp^	|*V*^bcp^|/*G*^bcp^
CAS(12 + *n*, 13)^a^	Th	0.061	0.151	1.244
	U	0.066	0.171	1.248
	Np	0.069	0.181	1.252
	Pu	0.072	0.192	1.253
CAS(*n*, 7)^a^	Th	0.027	0.039	1.428
	U	0.066	0.172	1.247
	Np	0.068	0.183	1.243
	Pu	0.072	0.193	1.252
core RAS^b^	Th	0.027	0.039	1.428
	U	0.066	0.172	1.247
	Np	0.068	0.183	1.243
	Pu	0.072	0.193	1.252
RKS/PBE0^c^	Th	0.062	0.139	1.318
	U	0.067	0.161	1.312
	Np	0.069	0.170	1.307
	Pu	0.071	0.173	1.327

a *n* = 0 (Th), 2 (U), 3 (Np), 4 (Pu).

b Core RAS and CAS(*n*, 7) are of identical quality w.r.t. the GS wavefunctions.

c Similar data are obtained with UKS/PBE0 (see Table S4).

The [AnCl_6_]^2−^ GS configurations originate from those of the An^4+^ ions, *i.e.* 5f^*n*^6d^0^ with *n* = 0, 2, 3, 4 for An = Th, U, Np, Pu, respectively, successively accommodating the increasing 5f occupation among the a_2u_ nonbonding and t_2u_ and t_1u_ antibonding orbitals in the highest possible spin multiplicity (Fig. 1). With PBE0, the lowest energy configuration is 5f^0^ for [ThCl_6_]^2−^, 5f^2^[t^2^_2u_] for [UCl_6_]^2−^, 5f^3^[t^3^_2u_] for [NpCl_6_]^2−^, and 5f^4^[a^1^_2u_t^3^_2u_] for [PuCl_6_]^2−^. Only [UCl_6_]^2−^ exhibits two low-lying configurations within less than 0.2 eV from the GS, namely 5f^2^[a^1^_2u_t^1^_2u_] and 5f^2^[a^1^_2u_t^1^_1u_]. The MO energy ordering for the GS configurations is depicted in Fig. 1. The 6d-based t_2g_ and e_g_ MOs occur around 7 and 10 eV respectively, and are destabilized slightly from Th to Pu. The 5f-based a_2u_, t_2u_ and t_1u_ orbitals are near-degenerate with 6d t_2g_ in [ThCl_6_]^2−^ but become strongly stabilized across the series. The stabilization is largest for a_2u_ and t_2u_, *i.e.* for the MOs that become occupied towards Pu. The trend is preserved with UKS/PBE0, although these calculations predict much larger stabilization of the a_2u_ and t_2u_ α-spin MOs towards Pu (Fig. S1†), and much larger 5f–3p mixing in the t_2u_ and t_1u_ α-spin MOs.

With MC-WFT, the SF electronic GS is the closed-shell HF configuration for [ThCl_6_]^2−^. The GS becomes multiconfigurational from [UCl_6_]^2−^ to [PuCl_6_]^2−^ (Table 1). The GS results obtained with the minimal CAS(*n*, 7) and with core-RAS, which comprises additionally the Cl 1s combinations and 6d t_2g_, are identical for all studied complexes (see Tables 1 and 2), as they should be. These results are overall similar to those predicted by the better correlated CAS(12 + *n*, 13) approaches. This aspect is particularly important since it shows that the core-RAS wavefunctions capture the GS electronic structure details to a similar degree to calculations with larger active spaces and therefore they can safely be used for the calculations and interpretation of the Cl K-edge XANES. In other words, the GS electronic structures arising from the same calculations used to generate the XANES spectra are sufficiently accurate, as shown by comparison with the GS calculations that use larger active spaces.

In the following, we focus on the valence electronic structure of the U, Np, and Pu complexes as predicted by core-RAS. The SF GS of [UCl_6_]^2−^ is threefold spatially degenerate, with 60% t^2^_2u_ and 35% a^1^_2u_t^2^_2u_ contributions (Table 1). This wavefunction composition is similar to that obtained by Su *et al.*^127^ with a (2, 7) active space, but it apparently differs from the single-configuration a^1.00^_2u_t^1.00^_1u_ GS wavefunction obtained by Beekmeyer and Kerridge with a very large active space.^128^ However, the isosurface plots of the bonding t_2u_ and t_1u_ MOs from our calculations (Fig. S3†) and those from ref. 128 are very similar, suggesting that the metal–ligand bonding interactions, which are ultimately important for rationalizing the chlorine K-edge spectra, are very similar in both sets of calculations. With SO coupling, an excited spin-singlet state mixes (10%) with the spin-triplet GS resulting in an orbitally nondegenerate SO GS with 90% contribution from the SF, spin-triplet GS.

Concerning [NpCl_6_]^2−^, the SF GS is spin-quartet A_2u_ dominated by the t^3^_2u_ configuration (>90%), apparently in agreement with the GS predicted by KST/PBE0. However, there is a very low-lying excited state at 0.08 eV, ^4^T_2u_, with a multiconfigurational wavefunction involving 59% t^2^_2u_t^1^_1u_ and 34% a^1^_2u_t^2^_2u_ (the one shown in Table 1). With dynamic correlation corrections of the energies, this state becomes the GS,^129^ but it remains a very low-energy excited state with our XPT2//core-RAS (0.03 eV) because of the imaginary shift needed to produce reliable core-ESs from these calculations (see Section 2). With PBE0, the t^2^_2u_t^1^_1u_ and a^1^_2u_t^2^_2u_ configurations are very high in energy, 3.40 and 1.87 eV, respectively, above the t^3^_2u_ GS. The PBE0 failure to predict the correct GS of [NpCl_6_]^2−^, or at least one of the contributing dominant configurations, stems from the single-determinant Kohn–Sham approach in combination with an approximate functional. The t^3^_2u_ configuration is the only one described qualitatively correctly by a single determinant, which apparently leads to a too strong stabilization with KST, compared to the alternatives. The question of the T_2u_*vs.* A_2u_ GS in the WFT calculations with and without dynamic correlation becomes irrelevant in the presence of SO coupling. No matter whether or not, and how, dynamic correlation is treated, the SO GS exhibits a strong admixture of different SF states, with largest contributions from the aforementioned ^4^T_2u_ (33%) and ^4^A_2u_ (20%).

The SF GS of [PuCl_6_]^2−^displays the largest multiconfigurational character by combining 44% a^1^_2u_t^1^_2u_t^2^_1u_, 39% a^1^_2u_t^3^_2u_ and 17% t^2^_2u_t^2^_1u_ configurations. Among these, the configuration a^1^_2u_t^3^_2u_ can be represented by a single determinant, and it represents the spin-free GS in the PBE0 calculations. A multitude of spin-quintet excited states occur below *ca.* 0.25 eV, which couple strongly with the GS quintet *via* the SO interaction. The SO GS is a pseudo-spin triplet, separated by only 0.13 eV from the next pseudo-triplet SO excited state, with only a minor contribution (15%) from the SF GS.

### 3.2 Ground-state metal–ligand bonding

The An–Cl BOs and metal 5f- and 6d shell populations (*n*_f_ and *n*_d_), derived from either NPA or Mulliken analyses of the SF GSs (Table 1), are far larger with RKS/PBE0 than with the CAS/RAS approaches, with steadily increasing discrepancies between the two approaches from Th to Pu particularly regarding the *n*_f_ and the BOs. For instance, in [PuCl_6_]^2−^, the excess *n*_f_, relative to Pu^4+^, of 1.37 (1.14) electrons with NPA (Mulliken) predicted by PBE0 is about twice as large as the excess *n*_f_ of 0.65 (0.46) predicted by core RAS. Although the differences between MC-WFT and KST are less pronounced for *n*_d_, the excess *n*_d_ is larger with PBE0 than with core RAS, by 0.20 (0.30). Note that KST/PBE0 with RKS (Table 1) *vs.* UKS (Table S3†) led to similar NPA and BO data. The differences between KST/PBE0 and the WFT approaches were expected to some degree: the present MC-WFT wavefunctions lack most of the dynamic correlations (the PT2 steps only correct the energies), and the active spaces do not correlate the bonding and antibonding t_2u_, t_2g_ and e_g_ orbitals. The latter seems to play no major role, however, because MC-WFT calculations for [UCl_6_]^2−^, with a very large active space correlating the An 5f, 6d orbitals and all the Cl 3p orbitals,^128^ gave a U–Cl BO [in the form of a delocalization index (DI) based on QTAIM analyses] of 0.44, not large and more in line with the BOs calculated here with CAS and core-RAS (0.65/0.75, Table 1) than with the PBE0 results. Moreover, ref. 128 reported B3LYP and PBE DIs of 0.63 and 0.62 respectively, also in line with the BOs from the present CAS/RAS calculations. It therefore appears that the KST/PBE0 calculations with STO basis sets overestimate the excess orbital populations and the BOs. In the following, we focus instead on bonding *trends* predicted by PBE0 *vs.* CAS/RAS, rather than on absolute values. The bonding trends turn out to be consistent (*vide infra*), and we found the same, with few exceptions, in a recent study of An(iii) complexes with a variety of ligands including chloride.^126^

Differences between KST/PBE0 and core-RAS can be seen, visually, in the isosurface plots of the t_2u_ and t_1u_ MOs shown in Fig. 1 (RKS), S1 (UKS) and S4 (core RAS).† With PBE0, there is pronounced AO mixing that appears along with the strong stabilization of these MOs going from Th to Pu. These MOs are more localized and contracted towards Pu in the core-RAS calculations. This aspect, however, is reflected only in the excess *n*_f_ from Mulliken population analysis, which decreases from Th to Pu in the CAS/RAS calculations but increases with KST/PBE0 (Tables 1 and S3†). In the NPA, the excess *n*_f_ population increases from Th to U and then decreases towards Pu with CAS/RAS (pronounced) and UKS/PBE0 (slightly). With RKS/PBE0, the excess *n*_f_ increases from Th to Pu, but the net increase from U to Pu (0.02) is negligible. The *n*_d_ values increase from Th to Pu with the MC-WFT approaches, specifically by 0.14 from U to Pu with NPA, suggesting a slight increasing trend in An 6d covalency. With RKS/PBE0, however, *n*_d_ increases only by 0.08 from Th to U in NPA, and then remains about the same toward Pu. The Mayer and Wiberg BOs are overall consistent regardless of the used approach, and differ only slightly, in particular from U to Pu. The WBO for instance, drops from U to Pu by 0.04 with RKS/PBE0, by 0.02 with UKS/PBE0, and by 0.05 with core-RAS. No clear variation is seen in the MBOs, which remain about the same from U to Pu (about 0.64 and 1.04 with MC-WFT and KST/PBE0 respectively). Additionally, we noted that the positive NPA metal charge is the lowest at U (Table S3†), in line with the NPA charge trend found in other complexes of the tetravalent actinides,^125^ underlining that there is likely more pronounced donation bonding in [UCl_6_]^2−^ compared to the other hexachlorides. Thus, the take-away from the NPA and BO analyses is that the An–Cl covalency peaks at [UCl_6_]^2−^ and remains about the same, perhaps slightly decreasing, towards [PuCl_6_]^2−^.

The pronounced An(5f)–Cl(3p) mixing in the t_2u_ and t_1u_ PBE0 MOs shown in Fig. 1 (RKS) and especially in Fig. S1 (UKS),† from [ThCl_6_]^2−^ to [PuCl_6_]^2−^, is in apparent contradiction with the NPA and BO data and therefore the AO mixing in these MOs does not reflect an increasing trend in 5f covalency going from Th to Pu. In line with the familiar concepts of MO theory, the steady energy stabilization of the An 5f shell along the An row leads to better energy matching with the Cl 3p shells in the KST/PBE0 calculations, such that AO mixing in the canonical KS MOs becomes large despite the somewhat decreasing AO overlap from Th to Pu. However, canonical AO mixing alone should not be equated with covalent bonding.^22,130–132^ For instance, in ref. 132, Sadhu and Dolg showed that energy-degeneracy driven AO mixing in frontier MOs of lanthanide(iii) and actinide(iii) complexes does not lead to stabilization of the systems if AO overlap is negligible. In the supplementary file of ref. 133 it is demonstrated by using a two-AO model system that the solutions of the generalized eigenvalue equation can become difficult to interpret in the weak-overlap near-degeneracy regime that is presumably reflected in the 5f bonding of actinides. The overlap of 5f with valence ligand orbitals decreases along the actinide row, but especially in the first half of the row it is definitely not negligible.^56,126^ Canonical AO mixing may occur for reasons unrelated to chemical bonding, for example because of matching Fock matrix elements (‘energies’), or when AO linear combinations form symmetry-adapted MOs. In order to obtain covalent bonding information, it is therefore best to use well-established measures such as the bond orders that we discussed already, QTAIM analyses, or orbital localization schemes.

**Fig. 2:**
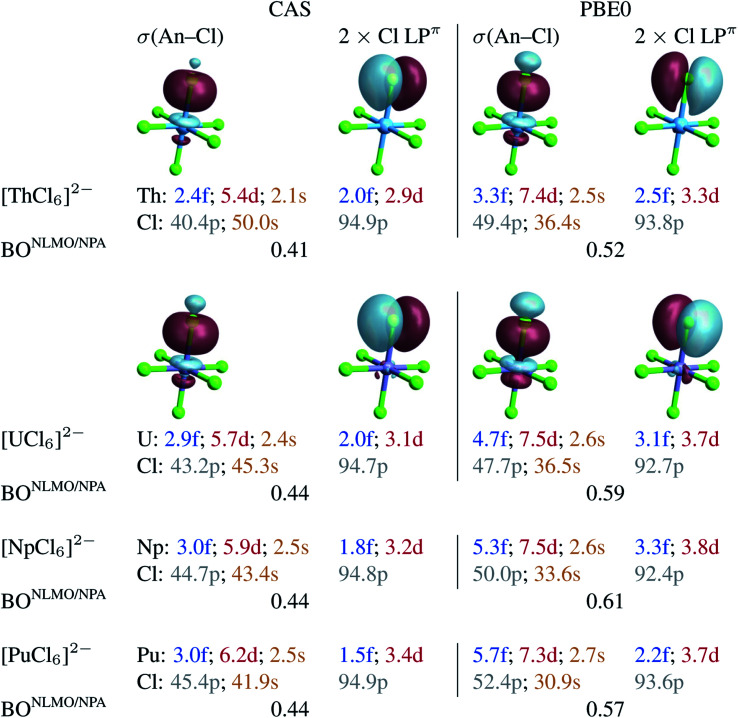
Selected NLMOs (±0.03 au isosurfaces) and their metal and ligand AO weight-% compositions representative of the An–Cl bonding interactions in [AnCl_6_]^2−^ (An = Th–Pu). Left panel: CAS(12 + *n*, 13) SF calculations. Right panel: RKS/PBE0 SF calculations (see Fig. S2† for UKS/PBE0 calculations). For the An = U–Pu complexes, the NLMOs shown are those of [PuCl_6_]^2−^. The weight-% data are given as averages over the six An–Cl bonds.

To capture the metal–ligand orbital mixing with contributions also from inactive orbitals, we subjected the spin-free CAS(12 + *n*, 13) GS densities to NBO calculations. For comparison, such calculations were also conducted with the KST/PBE0 GS densities. Natural localized molecular orbitals (NLMOs) are shown in Fig. 2. The key NLMOs obtained per An–Cl interaction describe a σ(An–Cl) bond and three Cl lone pairs, two of which display slight Cl → An π donation bonding character (Cl LP^π^) and one is a pure σ lone pair (Cl LP^σ^) pointing in the opposite direction as the An–Cl bond (not shown). The CAS-level NLMOs in Fig. 2 show highly polarized An–Cl σ bonds with a small increase in metal participation from Th (10%) to Pu (12%) in the σ(An–Cl) bond and no change in delocalization of the Cl LP^π^ over the metal (5%). Overall, there is a minor increase in the 5f weight in the σ(An–Cl) NLMOs, from 2.4% (Th) to 3.1% (Pu), counter-acted by a weak decrease of the 5f weights in the Cl LP^π^ NLMOs, from 2% (Th) to 1.5% (Pu). From U to Pu in particular, the total 5f contribution per An–Cl interaction decreases very slightly from 4.9% to 4.5%. Both the σ(An–Cl) and LP^π^ NLMOs show a very small increase in metal 6d contribution, from Th to Pu, by 0.8 and 0.5% respectively. From U to Pu, the total 6d contribution per An–Cl interaction increases very slightly by 0.8%. From U to Pu, the net Cl 3p contribution in the σ(An–Cl) NLMOs increases by about 2%, from 43.2 to 45.4%. This increase occurs at the expense of the Cl 3s contribution, and therefore there is no overall larger metal–ligand hybridization towards Pu. The small variations in the 5f and 6d contributions per An–Cl interaction are reflected in the calculated NLMO/NPA BOs based on the NLMO shared densities and bonding *vs.* antibonding overlap (given in Fig. 2), which increase but only slightly from Th (0.41) to U (0.44) and then remain the same towards Pu. This aspect is perfectly in line with the only weak variation of the An–Cl WBOs shown in Table 1. Thus, the NLMO analyses of the CAS densities do not indicate an overall increasing trend in An–Cl covalency from [ThCl_6_]^2−^ to [PuCl_6_]^2−^.

With PBE0, there is slightly larger metal participation in the σ(An–Cl) NLMOs, which increases from Th (13%, *vs.* 10% in CAS) to Pu (16%, *vs.* 12% in CAS). The net 5f contribution in these NLMOs follows an increasing trend, from 3.3% (Th) to 4.7% (U) to 5.7% (Pu). However, the net increase in metal 5f contribution per σ(An–Cl) NLMO, is only 2.4% from Th to Pu and only 1% from U to Pu in particular. The Cl LP^π^ donation bonds remain similarly polarized towards the metal from Th to Pu (∼6%), and the 5f net contribution in these delocalized lone pairs decreases slightly from U (3.1%) to Pu (2.2%). For each An–Cl interaction, and particularly from U to Pu, the total 5f contribution increases by 0.1%, from 7.8 to 7.9%, whereas the total 6d contribution decreases by 0.2% from 11.2 to 11.0%. As with CAS, in the σ(An–Cl) NLMOs, the chlorine 3p contribution increases from U to Pu, from 47.7 to 52.4%, but again at the expense of 3s contributions. The calculated NLMO/NPA BOs (Fig. 2) [0.52 (Th), 0.59 (U), 0.61 (Np), and 0.57 (Pu)] follow a similar trend overall as the PBE0 Mayer and Wiberg BOs shown in Table 1, and do not show a clear increasing trend in An–Cl covalency from Th to Pu, in agreement with the NLMO/CAS data. Similar conclusions are drawn from NLMO analyses of the UKS/PBE0 electronic structures (see Fig. S2†).

For comparison with the orbital-based bonding analysis, the [AnCl_6_]^2−^ GS densities obtained with PBE0 and CAS/RAS approaches were further subjected to QTAIM analyses. An assortment of bond metrics at the An–Cl bond critical points (BCPs) is shown in Table 2 (RKS/PBE0 *vs.* CAS/RAS) and Table S4† (UKS/PBE0). It has been pointed out that the presence of a BCP does not necessarily indicate a chemical bond, and there is a recommendation to use the term ‘line critical point’ (LCP) instead.^134,135^ We note this important detail but maintain the familiar BCP notation because in the [AnCl_6_]^2−^ series there are undoubtedly An–Cl bonding interactions. The QTAIM metrics are very similar particularly between PBE0, regardless of the used formalism, and CAS(12 + *n*, 13). The data based on the small CAS(*n*, 7) and core RAS are consistent with the data from the larger CAS, although with some deviations in the absolute values of the metrics predicted for [ThCl_6_]^2−^. In the latter case, the HF calculations [CAS(0, 7)] and core RAS are equivalent and deliver a more ionic picture of the metal–ligand bonding than the other calculations, as a result of over-localization of the electronic structure from lack of dynamic correlation. Both CAS(12 + *n*, 13) and PBE0 capture an increasing trend in the density at the BCP (*ρ*) from Th (0.061) to Pu (0.072), *i.e.* in this order there is a larger tendency of charge distribution in the An–Cl bonding region, thus some increase in covalency according to this measure. However, the Laplacian of the density (∇^2^*ρ*) becomes increasingly positive from Th to Pu (Table 2), showing a tendency of charge depletion at the BCP and enhancement of ionic character in this order. In the Nakanishi and Hayashi classification of weak and strong interactions,^136^ the An–Cl bonds are *regular* ionic. However, the An–Cl bonds are not classified as fully ionic by QTAIM, because the ratio between the potential energy density and the kinetic energy density at the BCP (|*V*|/*G*) is larger than 1 and shows an increasing trend from Th to Pu, *i.e.* the potential energy density dominates and electrons tend to localize at the BCPs. Thus, the QTAIM analyses of both CAS and PBE0 densities classify the metal–ligand bonds in [AnCl_6_]^2−^ as ‘mostly ionic with some covalency’. In other words, the dative bonds are strongly polarized toward Cl. QTAIM shows some inconsistency in that different metrics show ionicity or covalency to be increasing from Th to Pu. In agreement with the NPA and NLMO analyses, QTAIM does not identify a clear-cut trend of increasing donation bonding from [ThCl_6_]^2−^ to [PuCl_6_]^2−^, and we proceed in the next section by understanding how this conclusion is reflected in the chlorine K-edge XANES calculated with multiconfigurational approaches. Note that An^IV^ covalency that is not genuinely increasing in complexes with An ranging from Th/Pa–Pu has been assigned in other complex series as well, *e.g.* An(C_8_H_8_)_2_,^137,138^ An(C_5_H_5_)_4_,^139,140^ An(salen)_2_,^125^ and An(^Ar^acnac)_4_.^141^

We conclude this subsection by noting that the appearance of the core RAS t_2u_ and t_1u_ natural orbitals shown in Fig. S4,† becoming more localized and contracted towards Pu, reflect a slightly decreasing trend in 5f covalency. Large canonical mixing such as the one seen in the PBE0 Kohn–Sham MOs, can be reproduced in CAS calculations with sufficiently large active spaces, including both the bonding and antibonding t_2u_ and t_1u_ orbitals. Selected linear combinations among the two pairs of MOs will produce large AO mixing. However, by doing so the covalency in the system does not change because the configuration interaction coefficients will adapt to the change in the orbital basis and preserve the wavefunction of the system. The concept was illustrated initially by Mooßen and Dolg^142^ for the case of Ce(C_8_H_8_)_2_, and more recently by us^34^ for CeO_2_ and Ce(C_8_H_8_)_2_. Furthermore, it is important to note the following: in agreement with our *ab initio* data, the metal–ligand bonding extracted from the experimental XANES pre-edge intensities also shows that the total (5f + 6d) covalency per An–Cl interaction slightly increases from [ThCl_6_]^2−^ to [UCl_6_]^2−^ and then remains similar towards [PuCl_6_]^2−^.^56^ In ref. 56, Cl(3p) percentage contributions in the metal 5f-based and 6d t_2g_ MOs of [AnCl_6_]^2−^ were extracted based on a comparison with the intensity of the single pre-edge feature observed in *D*_2d_–Cs_2_CuCl_4_, following the lines of Solomon *et al.*^59^ The Cl 3p contributions to the 5f-based and 6d t_2g_ MOs, from the combined experimental pre-edge peak intensities of [AnCl_6_]^2−^, were 18, 21, 21 and 20% for An = Th, U, Np and Pu, respectively. That is, the experimental data show that the metal–ligand covalency increases from Th to U, and then remains similar towards Pu, in perfect agreement with our calculations. Even if something specific about the actinide complexes would prevent the extraction of numerically accurate Cl 3p percentages by direct comparison with Cs_2_CuCl_4_, the relative contributions within the [AnCl_6_]^2−^ series are reliable. For each An–Cl interaction, the experimental total Cl 3p contribution (8.1% (ref. 56)) in the 5f orbitals can be extracted only for the Pu complex according to our calculations (see Section 3.3), because only here are the transitions into 5f *vs.* 6d cleanly separated.

### 3.3 Cl K-edge XANES

**Fig. 3:**
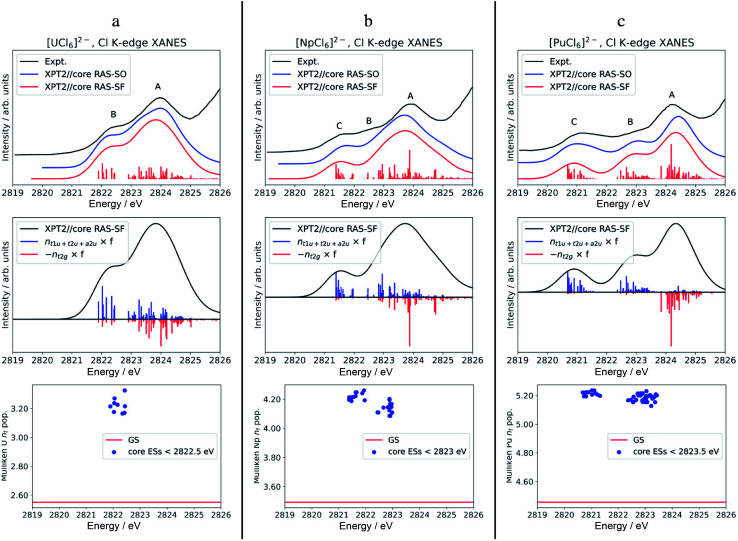
Calculated *vs.* experimental Cl K-edge XANES data in [AnCl_6_]^2−^ with An = U (panel a), Np (b), and Pu (c). Top row: Cl K-edge spectra. Center row: ‘stick’ spectra underlying the calculated Cl K edges are shown as oscillator strengths scaled by the population of An 5f (blue) *vs.* 6d (red) NOs in the excited state. For better visual separation, the 5f and 6d indicators are drawn in opposite directions. Bottom row: actinide *n*_f_ Mulliken population in the GS *vs.* low-energy core ESs with oscillator strength *f* > 10^−5^. Experimental spectra were digitized from the graphical material published in ref. 56.

The calculated Cl K-edge XANES with multiconfiguration XPT2//core RAS approaches, with and without SO coupling, is shown together with the experimental spectra^56^ in Fig. S5† for [ThCl_6_]^2−^ and Fig. 3 for [AnCl_6_]^2−^ (An = U–Pu). Note that our XANES calculations only target the pre-edge features below 2826 eV, which are due to transitions from Cl 1s into valence 6d t_2g_ and 5f a_2u_, t_2u_ and t_1u_ MOs.^55,56^ The main edges occurring slightly below 2828 eV, due to transitions from Cl 1s into valence 6d e_g_ MOs, among others, are therefore not covered in this study. Differences between the XPT2//core RAS-SF *vs.* RAS-SO spectra are insignificant for all the studied hexachlorides. Therefore, we focus on the spectra calculated without SO coupling in the following section.

The calculated pre-edges of the Cl K-edge XANES reproduce the experimental features very well, for all [AnCl_6_]^2−^ complexes. The agreement is encouraging especially for [NpCl_6_]^2−^ given that the spin-free GS of this system is incorrectly predicted by the XPT2/core RAS calculations (see Section 3.1). This outcome was expected, nevertheless, given that the 5f-based MOs and 6d t_2g_ are practically unchanged in a quartet A_2u_*vs.* a T_2u_ GS in [NpCl_6_]^2−^, and given that the Cl K-edge intensities are mainly sensitive to the 5f–3p mixing in the orbitals. There is a single peak A for [ThCl_6_]^2−^, a broader peak A with an emerging peak B at a lower energy for [UCl_6_]^2−^, and a broader peak A with two emerging peaks B and C at lower energies for [NpCl_6_]^2−^ and [PuCl_6_]^2−^. Peak labeling is provided in Fig. 3. The emergence of three pre-edge peaks when going from Th toward Pu is consistent with the PBE0 MO diagram shown in Fig. 1 (see also Fig. S1†), which shows that the possible accepting MOs for the core electron, 6d t_2g_ and 5f a_2u_, t_2u_, and t_1u_ are near-degenerate for Th whereas the 5f MOs drop in energy relative to 6d t_2g_, and split, moving from U to Pu. We emphasize that our pre-edge peak assignments with respect to core transitions into 5f and 6d t_2g_ valence MOs confirm the previous assignments.^55,56^ Note that in the WFT calculations for the U–Pu cases, both the GS and the intense core ESs are multiconfigurational, especially in the presence of SO coupling. This leads to the a_2u_, t_2u_ and t_1u_ MOs contributing to all relevant states such that their individual contributions to certain pre-edge peaks cannot be singled out. Thus, in the discussion of the spectra, we distinguish mainly between the overall contributions from 5f *vs.* 6d t_2g_.

#### 3.3.1 [ThCl_6_]^2−^

The calculated pre-edge consists of a single sharp peak derived from a threefold degenerate transition (part of the degenerate 1s → 6d t_2g_ grouping of transitions). The NO configuration of this intense core ES is t^0.76^_2g_5f^0.24^, with 5f including t_2u_ and t_1u_ contributions. The intensity, thus, arises mostly due to core electron transitions into 6d t_2g_. Visually, this aspect is depicted in the bottom panel of Fig. S5† which shows the ‘stick’ transitions weighted by the 6d t_2g_*vs.* 5f NO populations. The sizable 5f^0.24^ natural population in the intense core ES results from the near-degeneracy among the valence 5f-based MOs t_2u_ and t_1u_ and 6d t_2g_, *i.e.* the core ES wavefunction becomes a symmetry-adapted superposition of [Cl-1s_u_]6d^1^5f^0^ and [Cl-1s_g_]6d^0^5f^1^ (with u and g subscripts denoting *ungerade* and *gerade* symmetries, respectively) core-excited determinants. This near-degeneracy is also the reason why the Cl K-edge structure in [ThCl_6_]^2−^ shows no other pre-edge feature below ∼2824 eV. The absence of a second peak dominated by 5f excited state population is consistent with the experiment, because no such peak is resolved in the experimental spectrum.

#### 3.3.2 [AnCl_6_]^2−^, An = U–Pu

The origin of the two or three pre-edge features in the Cl K-edge XANES of [AnCl_6_]^2−^ with An = U–Pu is clearly visible in Fig. 3. The center row of the figure depicts the oscillator strengths under the spectral envelope weighted by the 6d t_2g_ (red sticks) *vs.* the combined 5f a_2u_, t_2u_, and t_1u_ (blue sticks) NO populations of the corresponding core ESs. For better visual separation, the scaled oscillator strengths for 6d and 5f are drawn in opposite directions (of course, negative signs should not be interpreted as an overall decreasing intensity).

For [UCl_6_]^2−^, the main pre-edge feature, peak A, is described by excitations between 2823 and 2825 eV. The lower energy group of excited states has primary configurations from transitions into 5f and secondary configurations from transitions into 6d t_2g_. The higher energy group of excited states has dominant contributions from transitions into 6d t_2g_, generating much of the peak intensity. The emergence of the new pre-edge peak B around 2822 eV is due to a grouping of core ESs derived almost exclusively from core transitions into the valence 5f-based MOs (blue sticks).

Beyond [UCl_6_]^2−^, the intense excitations under peak B and under the low-energy side of peak A move to lower energies and separate energetically to produce three peaks. Arriving at [PuCl_6_]^2−^ the ESs under Peak C, B, and A are now almost perfectly cleanly assigned to transitions into 5f, 5f, and 6d t_2g_, respectively. It therefore becomes apparent that, in terms of identity, the newly emerging peak along series U–Pu is actually peak B because it is formed by transitions into 5f that are grouped with transitions into 6d under peak A for [UCl_6_]^2−^. Note that for [NpCl_6_]^2−^, the peak B separation from peak A is slightly underestimated in the calculations, such that it appears as a weakly developed shoulder with the chosen broadening, rather than a weakly developed peak as in the experimental spectrum. Compared to [NpCl_6_]^2−^, the B and C peaks of [PuCl_6_]^2−^ are better separated from the main peak A, which follows the trend seen experimentally. Evidently, going from U to Pu, there is a clean separation of core transitions into the 5f-based MOs *vs.* 6d t_2g_ as the former strongly drop in energy. Pronounced multiplet effects are present and affect the spectral shapes over the whole pre-edge ranges.

It is tempting to rationalize the appearance of peaks B and C with respect to peak A, and particularly the intensity rise in peak C towards [PuCl_6_]^2−^, by an increase in An(5f)–Cl(3p) AO mixing in the acceptor MOs for the core electron. However, based on our analysis of the GS metal–ligand bonding in the WFT calculations, there is neither increased AO mixing in these MOs from U to Pu, nor is there an increasing trend in covalent bonding indicators such as BOs, AO populations, or QTAIM metrics. Particularly the NPA indicators, extracted from both WFT and KST calculations, indicate that the bonding across the series is more or less similar if not decreasing slightly (Section 3.2), despite that canonical mixing increases from U to Pu in some of the KST MOs. The evolution of the XANES spectrum from Th to Pu can be explained simply by depletion of intensity in peaks A and B, associated with the valence 5f-based MOs. These transitions accumulate under peak C as the energies of 5f MOs decrease. Likewise, the intensity decrease of peak B from U to Pu, relative to peak A, can be rationalized simply by the energetic separation of core ESs associated with transitions into 5f *vs.* 6d t_2g_, which is consistent with the energetic lowering of the 5f *vs.* 6d MOs. In other words, with the finding that the GS 5f/6d–3p covalency is quite similar across the series, redistribution of oscillator strengths among the peaks as the 5f-based MOs stabilize relative to 6d t_2g_ MOs which can fully explain the observed changes in relative pre-edge intensities moving from Th to Pu.

To address differences in the metal–ligand covalency in the GS *vs.* core ESs, we performed population analyses, and also tracked the QTAIM *ρ* metrics, for the most intense core ESs under peaks B and C. The core ES metal *n*_f_ data are displayed in Fig. 3, bottom row. The corresponding metal *n*_d_, Cl 3p and *ρ* are shown in Fig. S6.† The *n*_f_ data clearly highlight the one (U) *vs.* two (Np and Pu) groupings of transitions into 5f that form peaks B (U) and B & C (Np and Pu). Regardless of the complex, the metal *n*_f_ in the core ESs increase relative to the GS, on an average by 0.68 for U and Np, and by 0.74 for Pu. Thus, these core ESs under peaks B and C are essentially charge transfer states localizing most of the added valence electron density that results from the core excitation in the metal 5f shell. Slight electron redistribution occurs between 6d and Cl 3p in these core ESs of peaks B and C: there is an average decrease in the metal *n*_d_ population by 0.21 concomitant with an increase in the Cl 3p population by 0.13 relative to the GS (Fig. S6†). This apparent 6d–3p de-hybridization is captured by QTAIM analysis of the intense core ESs under peaks B and C, which show *ρ* at the BCP decreasing on an average by 0.005 au relative to the GS. In other words, in these excited states the metal–ligand covalency is weaker than that in the GS. Decreased covalency in the core ESs *vs.* the GS is an intuitive result, because the core ESs accommodate the core electron in MOs with antibonding character. The drop in *ρ* in the core ESs is rather small, however, and mostly associated with the decrease in 6d covalency. The reason for this is that the 5f covalency is not very strong in the GS, such that the population of the weakly antibonding 5f MOs in the core-ES does not trigger any notable orbital relaxation.

**Fig. 4:**
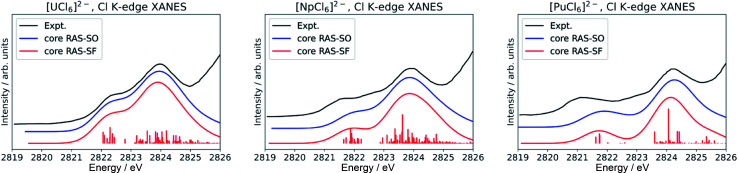
Calculated Cl K-edge XANES in [AnCl_6_]^2−^ (An = U–Pu) without including dynamic correlation effects. The experimental edges were digitized from ref. 56 and are given for comparison.

Finally, we note that inclusion of dynamic correlation is necessary to reach agreement with the experimental spectra particularly for the case of heavier [PuCl_6_]^2−^. With only core RAS-SF and core RAS-SO, *i.e.* without dynamic correlation, the Cl K-edge spectrum of [PuCl_6_]^2−^ does not exhibit the three-peak pre-edge pattern (Fig. 4).

## 4 Conclusions

The present study is part of an ongoing effort^34,40,42,74^ to establish protocols based on relativistic multiconfiguration wavefunction approaches for calculating XANES spectra in lanthanide and actinide complexes. Such calculations are able to establish the configurational admixture of the wavefunctions of the ESs that cause the intensity,^34^ and to analyze the chemical bonding and the involvement of the 5f shell individually in the GS and the relevant ESs. The valence electronic structure, metal–ligand bonding, and Cl K-edge XANES in [AnCl_6_]^2−^ (An = Th–Pu) complexes were investigated with scalar relativistic KST/PBE0 and multiconfigurational WFT approaches. In the latter, core-excitation RAS, a PT2 treatment of the dynamic correlation, and a state-interaction treatment of the SO coupling were employed. In particular, we focused on identifying and rationalizing An 5f covalency and its connection with pre-edge features appearing in the experimental Cl K-edge spectra.^56^

We found similar ground-state An 5f and 6d covalency in the complexes, in particular for U, Np, and Pu, with KST/PBE0 and different flavors of CAS/RAS calculations. The 5f-based a_2u_, t_2u_ and t_1u_ orbitals are strongly stabilized relative to the Cl 3p orbitals of matching symmetry going from Th to Pu. In the KST/PBE0 calculations, this goes along with increasing An(5f)–Cl(3p) AO mixing in some of the canonical MOs. However, the canonical mixing does not correlate with stronger donation bonding. For instance, the An–Cl Mayer, Wiberg and NLMO/NPA BOs, and electron donation to the An 5f AOs generally decrease from U to Pu, although only slightly. Furthermore, neither NLMO nor QTAIM analyses predict increased levels of covalency in [AnCl_6_]^2−^ for An = U, Np, Pu.

The Cl K-edge XANES of [AnCl_6_]^2−^ calculated with multiconfiguration WFT approaches agree very well with the experiments and show the emergence of two pre-edge peaks B and C at energies below the dominant pre-edge peak A when going from U to Pu. Pre-edge features B and C arise from transitions into the valence MOs with dominant 5f character that separate energetically from transitions into MOs with dominant 6d t_2g_ character along the series Th, U, Np, and Pu. For [PuCl_6_]^2−^, the spectrum is almost cleanly separated into intensity from the transitions into 5f (peaks C and B) *versus* transitions into 6d t_2g_ (peak A). Localized MO, bond order, and QTAIM analyses provide a consistent picture showing comparable 5f and 6d covalency for the set of complexes, and especially among U, Np, and Pu. Given the comparable involvement of 5f in donation bonding in [AnCl_6_]^2−^, as indicated by the calculations, and a lack of excited state orbital relaxation, the additional pre-edge peaks in the Cl K-edge XANES spectra from An = U–Pu are simply rationalized by the strong stabilization of the actinide 5f relative to 6d t_2g_ orbitals, which moves the intensity associated with transitions to 5f out of peak A and into peaks B and C. Multiplet effects are present too from [UCl_6_]^2−^ to [PuCl_6_]^2−^, and they generally cause broadening of the pre-edge features.

In agreement with the conclusions from the original work,^56^ our study shows that the appearance of the pre-edge peaks in the Cl K-edge XANES is incontrovertible evidence for 5f and 6d covalency in the An–Cl bonds. Both the experimental data and our calculations show that An 5f covalency increases slightly from Th to U, and remains the same or decreases very slightly from U to Pu. This is contrary to what one would assume based solely on the AO mixing in the 5f-based antibonding KS orbitals. MO theory favors AO mixing when the diagonal Fock matrix elements (‘energies’) match well for the metal and ligand AOs, which explains the increased 5f–3p mixing in the ground state 5f-based KS canonical MOs toward Pu, along with complementary mixing in lower-energy occupied MOs.

There is clearly sufficient overlap of 5f and ligand AOs in the earlier actinides such that canonical 5f mixing in the complexes ultimately also translates into covalent 5f bonding. The present calculations for [AnCl_6_]^2−^, however, also show that energetic near-degeneracy driven metal–ligand AO mixing in the antibonding 5f-based canonical MOs is not necessarily a quantitative indicator for the degree of covalent 5f bonding. The trend in the sizable^56^ 5f–3p overlap and the trend in the An(5f) and Cl(3p) Fock matrix element differences, which are both decreasing from Th to Pu, counterbalance each other, resulting in comparable 5f covalency that manifests itself in the localized MO analysis and other covalent bonding measures. The present calculations and the prior XANES experiments agree on the overall covalency trend. To rationalize the XANES spectra, there is no need to distinguish between the established concept of covalent bonding based on both AO overlap and AO mixing, *versus* an energy-degeneracy driven mixing mechanism without overlap.

## Data availability

Raw DFT and WFT outputs containing the data analyzed in the present article are available in a data repository hosted at Zenodo (DOI: 10.5281/zenodo.6091617).

## Author contributions

DCS and JA conceived the project, performed the theoretical calculations, analyzed the data, and wrote the manuscript. JA secured the funding for the research.

## Conflicts of interest

There are no conflicts of interest.

## Supplementary Material

SC-013-D1SC06454A-s001
