# Supplementary material for: Covalency in actinide(iv) hexachlorides in relation to the chlorine K-edge X-ray absorption structure
Source: Chem Sci. 2022 Feb 9;13(11):3194–207. doi: 10.1039/d1sc06454a (PMC8926251; doi:10.1039/d1sc06454a)
Supplement: SC-013-D1SC06454A-s001 [file SC-013-D1SC06454A-s001.pdf]

Electronic Supplementary Material (ESI) for Chemical Science.
This journal is © The Royal Society of Chemistry 2022

Electronic Supplementary Information for: Theoretical Study of Covalency in Actinide(IV) Hexachlorides in Relation to Chlorine K-Edge X-ray Absorption Structure

Dumitru-Claudiu Sergentu[a] and Jochen Autschbach[a,*]

[a]Department of Chemistry
University at Buffalo
State University of New York
Buffalo, NY 14260-3000, USA
email: jochena@buffalo.edu

November 18, 2021

Contents

S1 Additional computational details **S3**

List of Figures

S1 Unrestricted DFT/PBE0 calculations. Left panel: Qualitative MO diagram depicting the An 6d e_g and t_{2g} and 5f a_{2u}, t_{2u} and t_{1u} energy levels of $[AnCl_6]^{2-}$ (An = Th–Pu) systems. Right panel: Isosurface plots (±0.03 au) of the 5f t_{2u}, t_{1u} and 6d t_{2g} α-spin MOs with An–Cl antibonding character. S4

S2 Selected NLMOs (±0.03 au isosurfaces) and their An weight-% composition representative of the An–Cl bonding interactions in $[AnCl_6]^{2-}$ (An = Th–Pu). Left panel: CAS(12+*n*, 13) SF calculations. Right panel: UKS/PBE0 SF calculations. For the An = U–Pu complexes, the (α-spin) NLMOs shown are those of $[PuCl_6]^{2-}$. S5

S3 GS bonding t_{2u} and t_{1u} MOs (±0.03 au isosurfaces) of $[AnCl_6]^{2-}$ (An = Th–Pu). CAS(12+*n*, 13) calculations. S6

S4 GS 5f-based (antibonding) t_{2u} and t_{1u} MOs (±0.03 au isosurfaces) of $[AnCl_6]^{2-}$ (An = Th–Pu). Spin-free core RAS calculations. S7

S5 Upper figure: Calculated vs. experimental Cl K-edge XANES in $[ThCl_6]^{2-}$. Bottom figure: The 'stick' spectra underlying the calculated Cl K edge is shown as oscillator strengths scaled by the number of core-excited electrons into Th 5f (blue) and 6d (red) orbitals. S8
S6 Calculated vs. experimental Cl K-edge XANES data in $[AnCl_6]^{2-}$ with An = U (panel a), Np (b), and Pu (c). First row: Cl K-edge spectra. Second row: An n_d Mulliken population in the GS vs. low-energy core ESs with oscillator strength $f > 10^{-5}$. Third row: Cl 3p Mulliken population in the GS vs. low-energy core ESs with oscillator strength $f > 10^{-5}$. Fourth row: An–Cl ρ^{bcp} QTAIM metric in the GS vs. low-energy core ESs with oscillator strength $f > 10^{-5}$. Experimental spectra were digitized from graphical material published in Ref. 1. . . . . S9

List of Tables

S1 Gaussian broadening (σ, in eV) values used to generate the calculated Cl K-edge XANES spectra (XPT2//core RAS-SF and XPT2//core RAS-SO) and the energy shift (in eV) applied to align the calculated spectra with the experimental counterparts. S10
S2 Decomposition of the NPA f-shell total population (n_f) in terms of contributions from 5f and 6f AOs. S10
S3 UKS/PBE0 calculations. Metal f-shell (n_f) and d-shell (n_d) populations from NPA (Mulliken) analyses, NPA metal charge [q(An)], and Mayer / Wiberg bond order (MBO / WBO) for $[AnCl_6]^{2-}$ (An = Th–Pu). S11
S4 UKS/PBE0 QTAIM metrics calculated at the An–Cl bond critical points (BCPs) of the $[AnCl_6]^{2-}$ (An = Th–Pu) complexes. S11

S1 Additional computational details

Choice of active spaces and state interaction schemes.

Valence CAS calculations: For the open-shell $[AnCl_6]^{2-}$ (An = U–Pu) systems, the valence electronic structures are explored with CAS(n, 7) and CAS(12+n, 13) with n = 0 (Th, Hartree-Fock calculation), 2 (U), 3 (Np) and 4 (Pu). The correlated orbitals are the An 5f orbitals in CAS(n, 7), and additionally the 3 × Cl(3p) orbitals of t_{1u} symmetry (bonding counterparts of the 5f t_{1u} antibonding orbitals) + the 3 × An 6p orbitals in CAS(12+n, 13). CAS wavefunctions were calculated in separate state-average runs for all possible configurations per multiplicity block and per irreducible representation of the D_{2h} point group, i.e. 21 triplets and 28 singlets for $[UCl_6]^{2-}$, 35 quartets and 84 doublets for $[NpCl_6]^{2-}$, and 35 quintets, 210 triplets and 196 singlets for $[PuCl_6]^{2-}$.

Core RAS calculations: Ligand K-edge XANES was calculated with the core RAS approach following a similar strategy and active space selection as in our previous work on various cerium and actinide complexes.[2–4] In these calculations, the six Cl $1s^2$ orbitals with at most one electron-hole spanned RAS1, the seven actinide $5f^n$ orbitals ($1 \times a_{2u}$, $3 \times t_{1u}$, $3 \times t_{2u}$ in O_h) spanned RAS2, and the three actinide 6d orbitals ($3 \times t_{2g}$ in O_h) with at most one electron occupation spanned RAS3. Since we focused on the description of the Cl K-edge XANES below ∼2824 eV, known to be generated by core transitions into the 5f and 6d t_{2g} valence orbitals,[1,5] the remaining two 6d e_g orbitals were not included in the active spaces. For each $[AnCl_6]^{2-}$ complex, all electric-dipole-allowed core excited states (ESs) were calculated in separate state-average runs per any possible spin-multiplicity block and per D_{2h} irrep. Due to technical reasons related to degeneracies breaking with the CAS second-order perturbation theory (CAS-PT2), dynamic correlation was introduced with a series of individual single-state single-reference extended multistate (XMS) PT2[6] (XPT2) calculations for each state, or group of degenerate states. The XPT2 calculations used an IPEA shift of zero and a very large imaginary shift (IMAG) of 2 Hartree in order to eliminate intruder states and to improve the similarity in the reference weights between the valence states and the core ESs.

Core RAS + RASSI: For $[ThCl_6]^{2-}$ and $[UCl_6]^{2-}$, all the caluclated core ESs were used in restricted active space state interaction (RASSI) calculations to compute SO couplings and electric-dipole oscillator strengths. Since the number of core ESs is too large, RASSI calculations were performed with a reduced number of core ESs (determined by convergence of the Cl K edge XANES) in the cases of $[NpCl_6]^{2-}$ and $[PuCl_6]^{2-}$. Concerning $[NpCl_6]^{2-}$, all spin-sextet core ESs were retained in RASSI, plus 201 spin-doublet and 201 spin-quartet ESs per each *gerade* irrep of D_{2h}. Concerning $[PuCl_6]^{2-}$, all spin-septet core ESs were retained in RASSI plus 201 spin-quintet, 201 spin-triplet and 201 spin-singlet core ESs per each *ungerade* irrep of D_{2h}. For all $[AnCl_6]^{2-}$ complexes, in addition to the core ESs, all valence states of all possible spin-multiplicities arising from An^{IV} $5f^n$ were retained in RASSI.

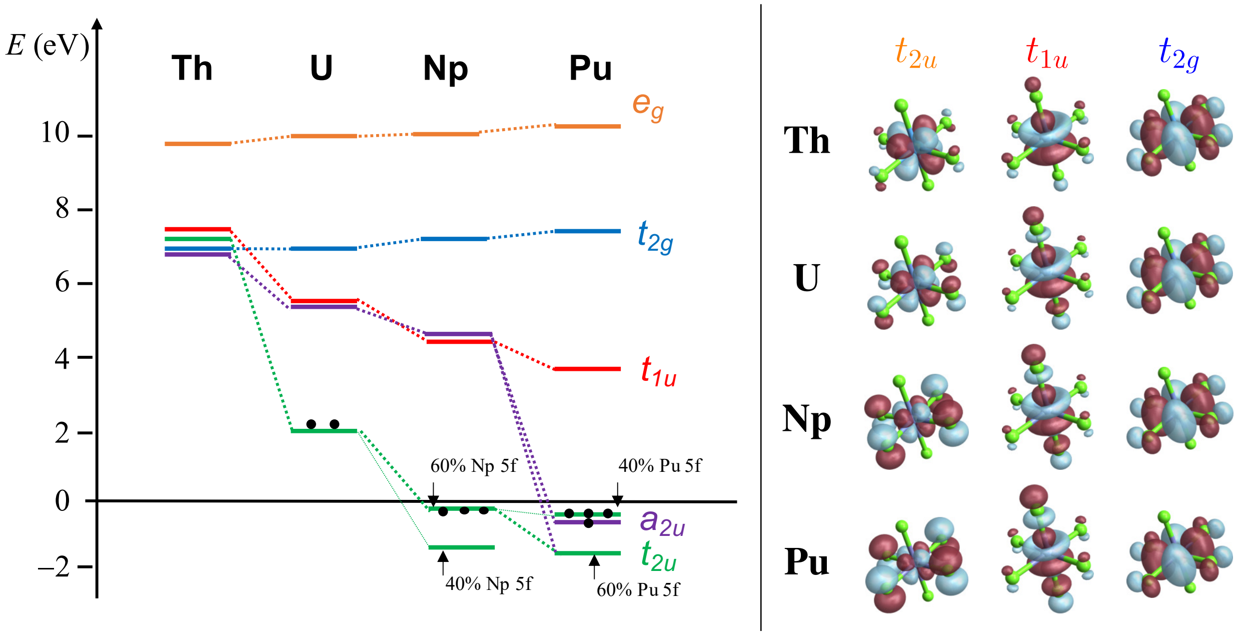

Figure S1: Unrestricted DFT/PBE0 calculations. Left panel: Qualitative MO diagram depicting the An 6d e_g and t_{2g} and 5f a_{2u}, t_{2u} and t_{1u} energy levels of $[AnCl_6]^{2-}$ (An = Th–Pu) systems. Right panel: Isosurface plots (±0.03 au) of the 5f t_{2u}, t_{1u} and 6d t_{2g} α-spin MOs with An–Cl antibonding character.

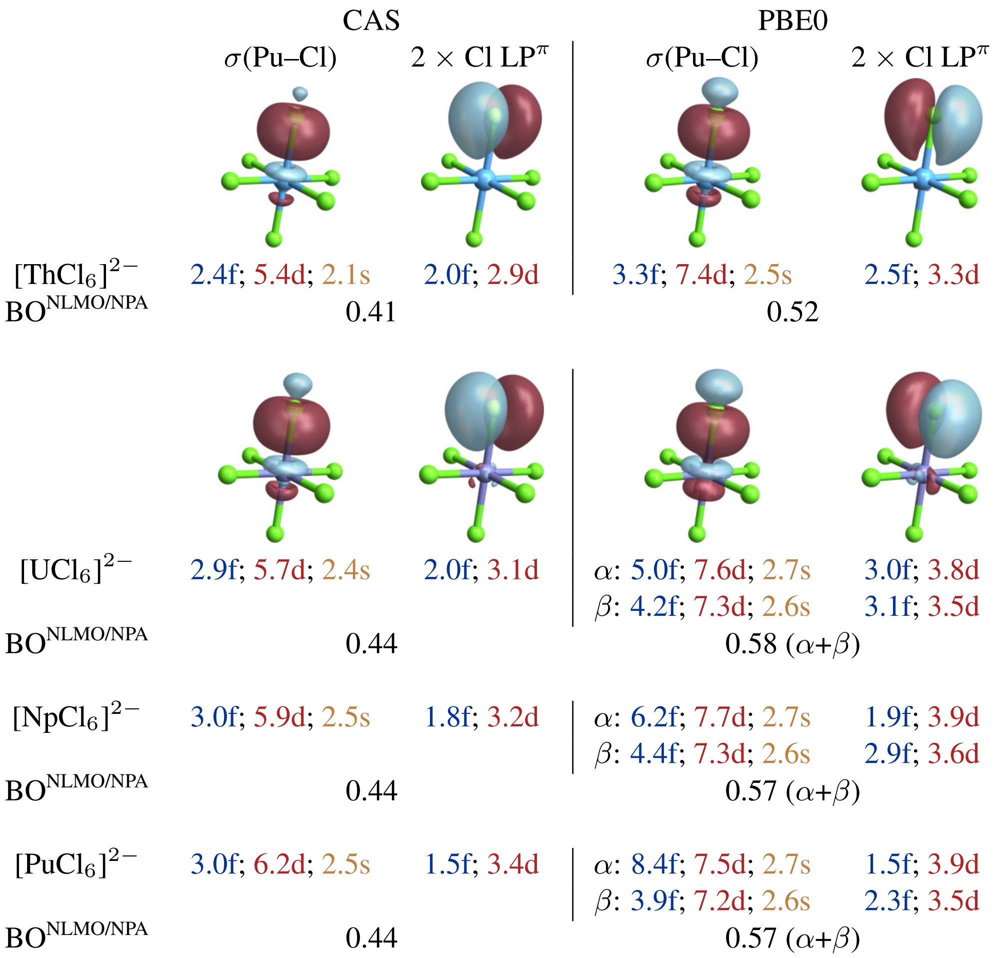

Figure S2: Selected NLMOs (±0.03 au isosurfaces) and their An weight-% composition representative of the An–Cl bonding interactions in $[AnCl_6]^{2-}$ (An = Th–Pu). Left panel: CAS(12+n, 13) SF calculations. Right panel: UKS/PBE0 SF calculations. For the An = U–Pu complexes, the (α-spin) NLMOs shown are those of $[PuCl_6]^{2-}$.

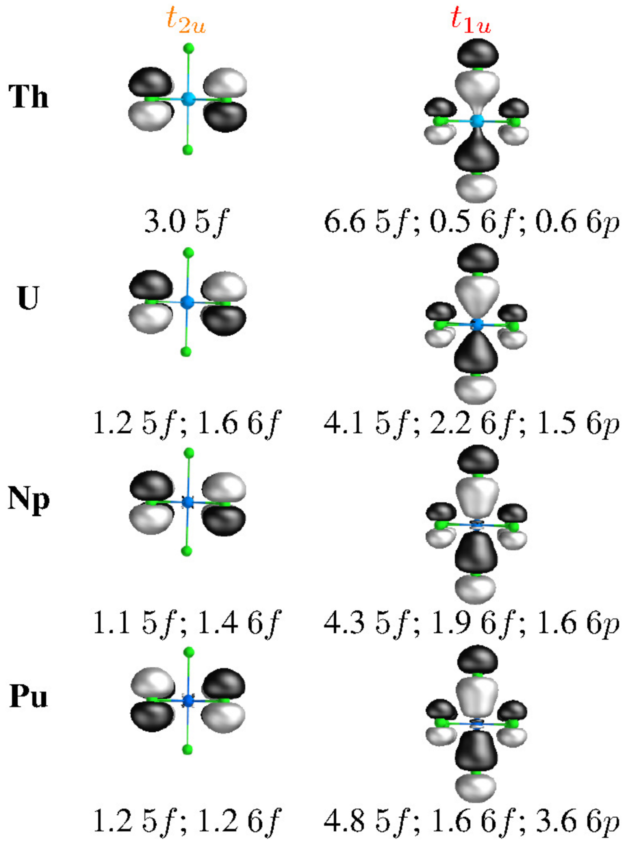

Figure S3: GS bonding t_{2u} and t_{1u} MOs (±0.03 au isosurfaces) of $[AnCl_6]^{2-}$ (An = Th–Pu). CAS(12+n, 13) calculations.

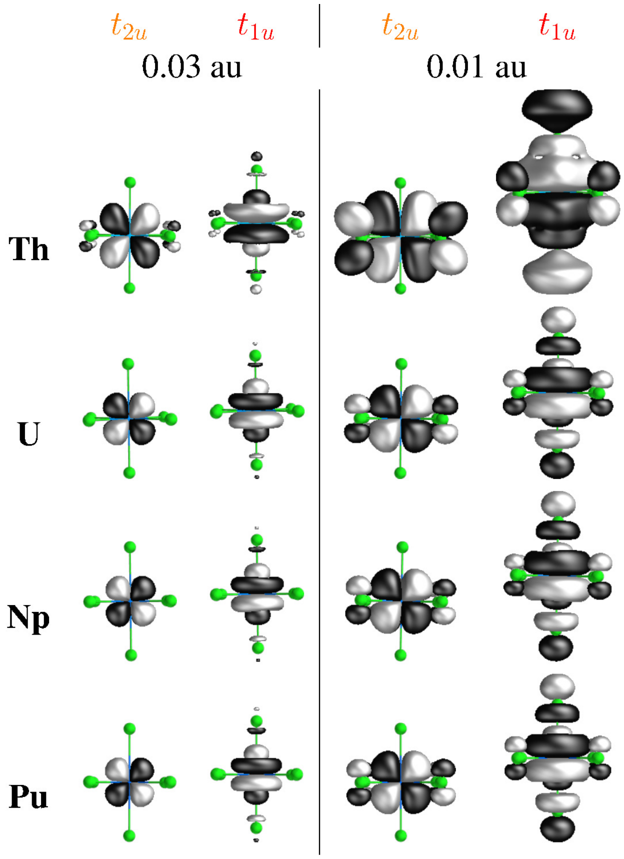

Figure S4: GS 5f-based (antibonding) t_{2u} and t_{1u} MOs (±0.03 au isosurfaces) of $[AnCl_6]^{2-}$ (An = Th–Pu). Spin-free core RAS calculations.

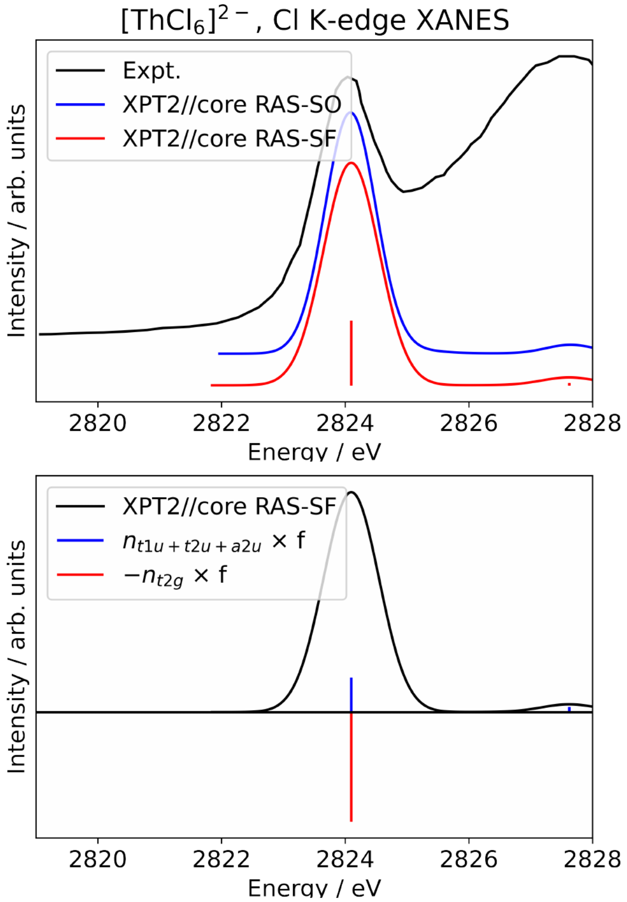

Figure S5: Upper figure: Calculated vs. experimental Cl K-edge XANES in $[ThCl_6]^{2-}$. Bottom figure: The 'stick' spectra underlying the calculated Cl K edge is shown as oscillator strengths scaled by the number of core-excited electrons into Th 5f (blue) and 6d (red) orbitals.

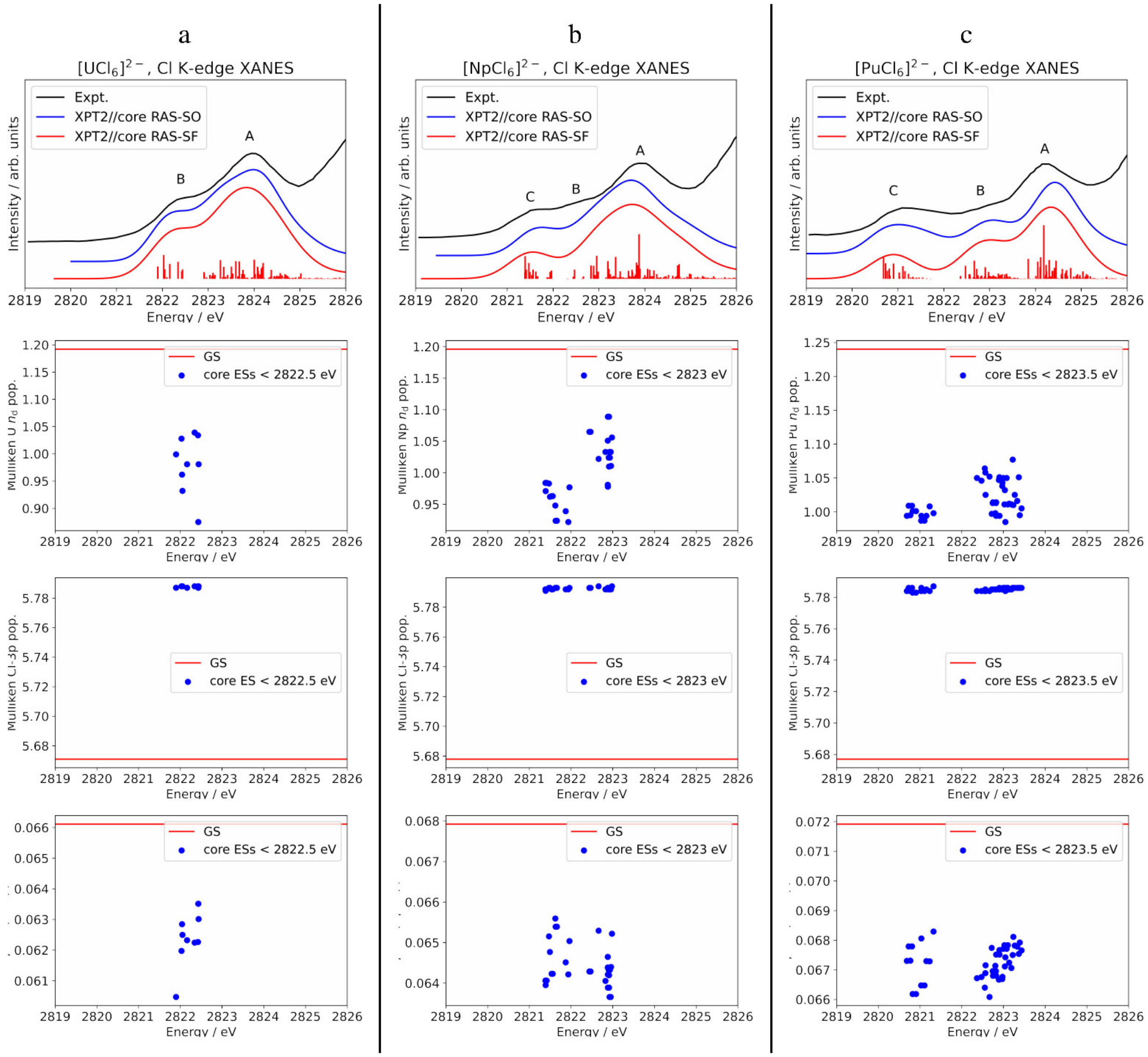

Figure S6: Calculated vs. experimental Cl K-edge XANES data in $[AnCl_6]^{2-}$ with An = U (panel a), Np (b), and Pu (c). First row: Cl K-edge spectra. Second row: An n_d Mulliken population in the GS vs. low-energy core ESs with oscillator strength $f > 10^{-5}$. Third row: Cl 3p Mulliken population in the GS vs. low-energy core ESs with oscillator strength $f > 10^{-5}$. Fourth row: An–Cl ρ^{bcp} QTAIM metric in the GS vs. low-energy core ESs with oscillator strength $f > 10^{-5}$. Experimental spectra were digitized from graphical material published in Ref. 1.

Table S1: Gaussian broadening (σ, in eV) values used to generate the calculated Cl K-edge XANES spectra (XPT2//core RAS-SF and XPT2//core RAS-SO) and the energy shift (in eV) applied to align the calculated spectra with the experimental counterparts.

	$[ThCl_6]^{2-}$	$[UCl_6]^{2-}$	$[NpCl_6]^{2-}$	$[PuCl_6]^{2-}$
σ (SF / SO)	0.45 / 0.4	0.45 / 0.4	0.45 / 0.4	0.45 / 0.4
Shift (SF / SO)	20.35 / 20.35	20.3 / 20.3	20 / 20	19.9 / 20.2

Table S2: Decomposition of the NPA f-shell total population (n_f) in terms of contributions from 5f and 6f AOs.

An	Approach	5f	6f	n_f
Th	CAS(12, 13)	0.76	0.04	0.80
	HF	0.75	0.03	0.78
	core RAS	0.75	0.03	0.78
	RKS/PBE0	1.00	0.03	1.03
U	CAS(14, 13)	2.43	0.46	2.89
	CAS(2, 7)	2.39	0.46	2.85
	core RAS	2.39	0.46	2.85
	PBE0	3.04	0.31	3.35
Np	CAS(15, 13)	3.42	0.37	3.79
	CAS(3, 7)	3.39	0.37	3.76
	core RAS	3.39	0.37	3.76
	RKS/PBE0	4.15	0.21	4.36
Pu	CAS(16, 13)	3.45	0.23	4.68
	CAS(4, 7)	4.41	0.24	4.65
	core RAS	4.41	0.24	4.65
	RKS/PBE0	5.26	0.11	5.37

Table S3: UKS/PBE0 calculations. Metal f-shell (n_f) and d-shell (n_d) populations from NPA (Mulliken) analyses, NPA metal charge [q(An)], and Mayer / Wiberg bond order (MBO / WBO) for $[AnCl_6]^{2-}$ (An = Th–Pu).

An	n_f	n_d	MBO / WBO	q(An)
Th	1.03 (0.91)	1.70 (1.55)	0.99 / 0.89	0.904
U	3.26 (3.04)	1.78 (1.60)	1.03 / 0.96	0.515
Np	4.23 (4.02)	1.81 (1.61)	1.05 / 0.96	0.563
Pu	5.22 (5.07)	1.79 (1.56)	1.05 / 0.94	0.588

Table S4: UKS/PBE0 QTAIM metrics calculated at the An–Cl bond critical points (BCPs) of the $[AnCl_6]^{2-}$ (An = Th–Pu) complexes.

An	ρ^{bcp}	$\nabla^2\rho^{bcp}$	$\|V^{bcp}\|/G^{bcp}$
Th	0.062	0.139	1.318
U	0.067	0.160	1.313
Np	0.069	0.164	1.326
Pu	0.072	0.165	1.350

References

[1] J. Su, E. R. Batista, K. S. Boland, S. E. Bone, J. A. Bradley, S. K. Cary, D. L. Clark, S. D. Conradson, A. S. Ditter, N. Kaltsoyannis, J. M. Keith, A. Kerridge, S. A. Kozimor, M. W. Löble, R. L. Martin, S. G. Minasian, V. Mocko, H. S. L. Pierre, G. T. Seidler, D. K. Shuh, M. P. Wilkerson, L. E. Wolfsberg and P. Yang, *J. Am. Chem. Soc.*, 2018, **140**, 17977–17984.

[2] D.-C. Sergentu, T. J. Duignan and J. Autschbach, *J. Phys. Chem. Lett.*, 2018, **9**, 5583–5591.

[3] G. Ganguly, D.-C. Sergentu and J. Autschbach, *Chem. Eur. J.*, 2020, **26**, 1776–1788.

[4] D.-C. Sergentu, C. H. Booth and J. Autschbach, *Chem. Eur. J.*, 2021, **27**, 7239–7251.

[5] S. G. Minasian, J. M. Keith, E. R. Batista, K. S. Boland, D. L. Clark, S. D. Conradson, S. A. Kozimor, R. L. Martin, D. E. Schwarz, D. K. Shuh, G. L. Wagner, M. P. Wilkerson, L. E. Wolfsberg and P. Yang, *J. Am. Chem. Soc.*, 2012, **134**, 5586–5597.

[6] T. Shiozaki, W. Györffy, P. Celani and H.-J. Werner, *J. Chem. Phys.*, 2011, **135**, 081106.
